# Hepatic elastin content is predictive of adverse outcome in advanced fibrotic liver disease

**DOI:** 10.1111/his.13499

**Published:** 2018-04-16

**Authors:** Timothy J Kendall, Grace E Dolman, Catherine M Duff, Emma C Paish, Abed Zaitoun, William Irving, Jonathan A Fallowfield, Indra N Guha

**Affiliations:** ^1^ Division of Pathology University of Edinburgh Edinburgh UK; ^2^ MRC Centre for Inflammation Research University of Edinburgh Edinburgh UK; ^3^ NIHR Nottingham Biomedical Research Centre (BRC) Nottingham University Hospitals NHS Trust and the University of Nottingham Nottingham UK; ^4^ MRC Human Genetics Unit Institute of Genetics & Molecular Medicine University of Edinburgh Edinburgh UK

**Keywords:** cirrhosis, elastin, hepatitis C virus, prognosis

## Abstract

**Aims:**

The aim of this study was to determine if elastin content in needle core native liver biopsies was predictive of clinical outcome in patients with chronic hepatitis C virus‐related chronic liver disease.

**Methods and results:**

Elastin contents in liver biopsies were determined by image analysis, technically validated in an independent centre, and correlated with outcome in patients with advanced (Ishak stage ≥5) chronic hepatitis C virus‐related chronic liver disease. Elastin was robustly quantified in an operator‐independent and laboratory‐independent manner, with very strong correlation of elastin staining measured with two methods of image classification (*r*_s_ = 0.873, *P* < 0.00001). Elastin content (but not absolute scar content or Ishak stage) was predictive for future clinical outcomes. In a cohort of patients without sustained virological response, the median hepatic elastin content was 3.4%, and 17 patients (57%) progressed to a liver‐related clinical outcome; 11 of the 15 patients (73%) with a hepatic elastin content of >3.4% progressed to a clinical outcome, as compared with only six of 15 (40%) with an elastin content of <3.4%. The difference in time to outcome was significant.

**Conclusions:**

We describe a simple and reproducible method for elastin quantification in liver biopsies that provides potentially valuable prognostic information to inform clinical management.

## Introduction

Although the safety of needle biopsy of the liver is increasing,^1^ there remain non‐zero mortality and morbidity rates, even with image‐guided procedures,^2^ and biopsy remains essential for the diagnosis and assessment of liver disease.^3^ However, in contrast to the use of biopsy material from other tissues,^4^ additional evaluation to yield prognostic, personalised information is not undertaken.

Chronic liver disease (CLD) represents a significant global health burden. In progressive liver injury, fibrotic neomatrix production, coordinated by hepatic myofibroblasts, exceeds native matrix degradation by matrix metalloproteinases (MMPs), resulting in progressive scar accumulation. However, after cessation of injury or treatment of the primary disease, the liver has the capacity for profound recovery. Data from well‐characterised animal models^5^, ^6^ and studies in human CLD^7^, ^8^ have demonstrated the potential reversibility of hepatic fibrosis. Moreover, histological regression of fibrosis is associated with a reduction in portal hypertension and improved clinical outcomes.^9^, ^10^, ^11^ The factors limiting the reversibility of fibrosis are less well understood, particularly in human CLD. Such factors could be important in predicting future decompensation events (e.g. variceal bleeding and ascites) or persistent hepatocellular carcinoma (HCC) risk,^12^, ^13^ and in the selection of patients for potential antifibrotic trials.

Elastin is an extracellular matrix (ECM) protein conferring elastic recoil to tissues. It is extremely stable *in vivo*, owing to cross‐linking and extreme hydrophobicity. Elastin is only a minor ECM component in normal liver, but it is actively synthesised as its soluble precursor, tropoelastin, by hepatic myofibroblasts in human fibrotic liver.^14^ Covalent cross‐linking of tropoelastin monomers results in an insoluble, mature elastin polymer that makes accumulated scar ECM more resistant to degradation, limiting the reversibility of fibrosis.

Evidence for elastin turnover *in vivo* comes from studies showing elevated serum levels of MMP‐mediated elastin breakdown fragments^15^ and urinary concentrations of markers of degradation of mature cross‐linked elastin (desmosine and isodesmosine) in patients with cirrhosis.^16^ Furthermore, these urinary biomarkers correlated with liver fibrosis scores in biopsies from patients with CLD secondary to hepatitis C virus (HCV) infection and alcohol consumption. Elastin turnover has also been studied longitudinally in liver biopsies from 21 patients with chronic viral hepatitis;^17^ this indicated that deposition of elastic fibres occurred concomitantly with the formation of thick collagen bands. This is consistent with other studies showing that older scars in liver biopsy specimens can be identified by their elastin content.^18^, ^19^, ^20^ More recently, hepatic elastin content has been shown to be associated with subsequent progression to the development of HCC in a cohort of patients with advanced fibrosis related to HCV.^21^

We hypothesised that the elastin content of fibrotic ECM in advanced CLD varies between individuals, and that hepatic elastin content may predict the occurrence of adverse clinical events. We have developed a robust, reproducible method that utilises existing biopsy material to quantify hepatic elastin and predict poor clinical outcome. Extracting additional information from biopsy material also favourably shifts the risk/benefit ratio of the procedure.

## Materials and methods

### Patient cohorts

#### Trent HCV biopsies

The study cohort was derived from a single centre (Nottingham) within the Trent Study of Patients with Hepatitis C Virus Infection, a prospective observational study designed to follow the natural history of HCV infection.^22^ Patients underwent liver biopsy as part of routine clinical care, and consented for tissue surplus to diagnostic purposes to be used for research. Albumin–bilirubin scores were calculated to assess the severity of liver dysfunction.^23^ The Trent HCV study was approved by the regional ethics committee (MREC 98/3/55).

To enrich the study for a future endpoint of clinical outcomes, we selected patients who had both advanced liver fibrosis and evidence of progressive disease. The inclusion criteria for elastin evaluation were as follows: active chronic HCV infection without a sustained virological response to therapy before or after biopsy; biopsy performed before 2011, allowing time for clinical outcomes to develop; biopsy assessed as Ishak stage ≥5 by an independent histopathologist blinded to other clinical information; adequate tissue remaining in the block available for elastin immunohistochemistry; and no clinical outcome before biopsy.

#### Lothian explant study samples

For technical validation of elastin staining by the use of samples from a different centre stained in an independent laboratory, non‐hilar sections from human explant liver were obtained by application to the Lothian NRS Human Annotated Bioresource (ethical review number 15/ES/0094). Samples were of mixed aetiology: seven cirrhotic (two alcohol‐related liver disease, one primary sclerosing cholangitis, two primary biliary cholangitis, one HCV, and one cryptogenic) and one non‐fibrotic (acute liver failure without fibrosis, attributed to drug‐induced liver injury).

### Histopathology

#### Quantitative histology and digital image analysis using the Trent HCV study biopsies

Four‐micrometre sections from biopsies were stained with picrosirius red (PSR), as described previously,^5^ for Ishak^24^ and Laennec^25^ scoring and quantification of liver fibrosis.

To identify elastin, 4‐μm sections were stained with a commercially available rabbit IgG polyclonal antibody against elastin (ab21610; Abcam, Cambridge, UK). The primary antibody was used at a dilution of 1:200 (15 min) on Leica Bond Max stainers after antigen unmasking with Epitope retrieval solution 2 (EDTA‐based pH 9.0, AR9640; Leica Microsystems, Milton Keynes, UK). Staining was visualised with the Bond Polymer Refine Detection kit (DS9800; Leica Microsystems).

For biopsies from the Trent HCV study cohort, whole‐slide images of PSR‐stained sections and sections stained for elastin were acquired at ×20 magnification (NanoZoomer; Hamamatsu Photonics, Shizuoka, Japan) and split manually into smaller tiles. Post‐acquisition analysis was performed with an imagej 26 plugin created in‐house, employing statistical colour modelling to threshold images.^27^ No manual curation or masking of structures was undertaken before analysis.

Quantification of elastin immunopositivity was repeated independently at a second centre with the same raw whole‐slide images. Images were split by the use of ndpisplit 28 into tiles of ×5 magnification before the application of a classifier that had been generated by a specialist liver histopathologist using the machine learning weka plugin in fiji.^29^, ^30^ All analysis was undertaken blind to all clinical and histological data.

#### Lothian liver explant samples

Additional work was undertaken at a second UK liver centre with alternative non‐automated staining protocols. Four‐micrometre sections of explanted cirrhotic liver were stained both with (Verhoeff's) Elastic van Gieson (EVG) and the same anti‐elastin primary antibody at the same dilution (1:200). Antigen retrieval was undertaken by 15 min of microwaving in EDTA pH 9.0 solution; the primary antibody was applied for 1 h at room temperature; and signal amplification was performed with a VECTASTAIN ABC HRP kit (Vector Laboratories, Peterborough, UK), according to the manufacturer's instructions.

### Clinical outcomes

A clinical outcome was defined as the first event recorded of: (i) ascites requiring treatment; (ii) variceal bleeding; (iii) overt hepatic encephalopathy; (iv) orthotopic liver transplantation; (v) liver‐related death; or (vi) development of HCC. Patients presenting with new‐onset ascites but diagnosed with HCC during investigation were recorded as having HCC. Data collection ceased in 2014, and none of the patients received treatment with direct‐acting antiviral agents. Data from hospital records were supplemented with data from the Office of National Statistics on cause of death and cancer registrations. Patients who did not reach a clinical outcome during the follow‐up period were censored at either the time when they were last seen alive without evidence of a liver‐related clinical outcome, or the time of non‐liver‐related death.

### Statistics

All data were tested for normality with the Shapiro–Wilk test and by examination of Q–Q plots, allowing appropriate parametric or non‐parametric tests to be used. Parametric data are described as mean ± standard deviation; non‐parametric data are presented as median with interquartile range (IQR). Spearman's correlation coefficient (*r*_s_) was used to measure the strength and direction of association between two ranked non‐parametric variables. Bland–Altman (BA) plots and intraclass correlation (ICC) coefficients were used for additional comparison of elastin quantification methods. The difference in time to outcomes was assessed with the log‐rank (chi‐square) test. Cox regression analysis was used to determine factors associated with time to clinical outcomes. Statistical analysis was performed with IBM spss statistics 22 and the rstudio implementation of r.^31^ A *P*‐value of <0.05 was considered to be statistically significant.

## Results

### Development, technical validation and reproducibility of elastin staining

**Figure 1:**
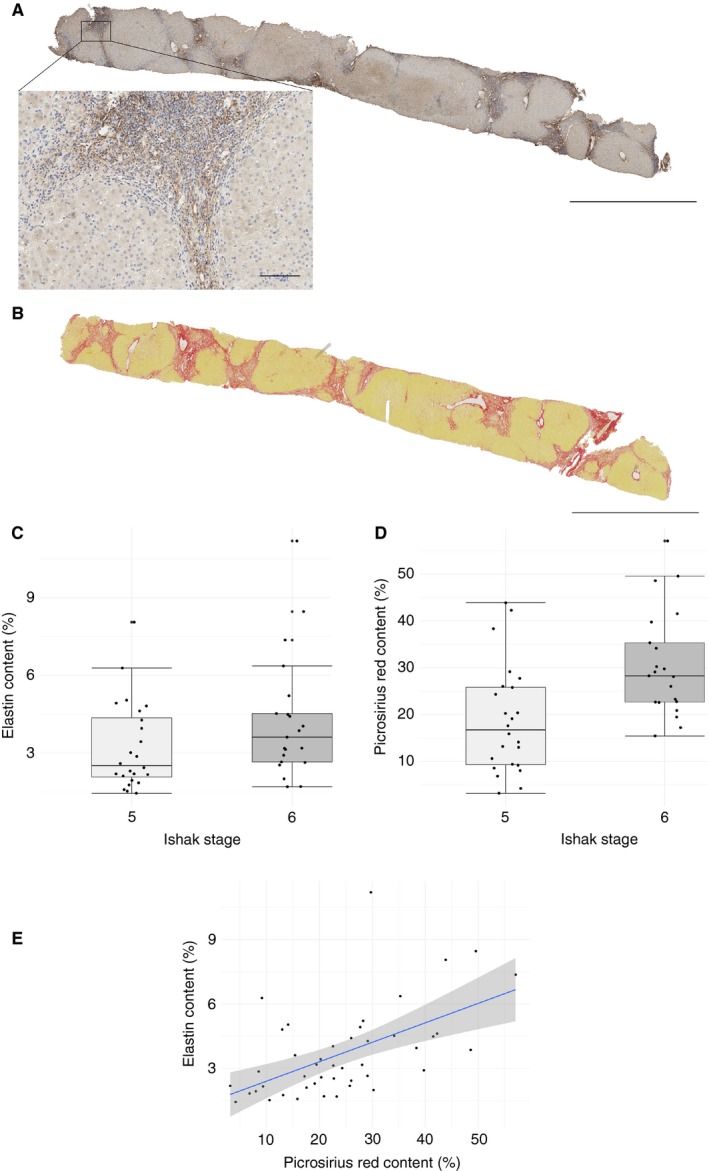
Quantification of elastin and fibrosis in biopsies of advanced (Ishak stage ≥5) hepatitis C virus infection. Biopsies from patients with chronic hepatitis C virus infections and advanced stage (Ishak stage ≥5) were stained with an antibody against elastin or with picrosirius red (PSR); representative whole‐slide images of a single case are shown (**A** and **B**, respectively; main scale bars 5 mm, inset 100 μm). Elastin and PSR contents were quantified by image analysis; there was no significant difference in elastin content between Ishak stages 5 and 6 (**C**, 2.51%, interquartile range (IQR) 2.07–4.36, and 3.61%, IQR 2.65–4.52, respectively; independent samples median test, *P* = 0.27] but there was a significant difference in PSR content between Ishak stages 5 and 6 (**D**, 18.80% ± 11.52% and 30.57% ± 11.24%, respectively, Welch two‐sample *t*‐test, *P* = 0.0012). There was a moderate positive correlation between elastin content and collagen content (**E**,* r*_s_ = 0.58, *P* = 0.000047, regression line from fitted linear model and 95% confidence intervals).

Elastin and PSR contents in the Trent HCV study samples were quantified with the previously described imagej plugin^27^ (Figure 1A,B); patient characteristics of the cohort are shown in Table 1. The median elastin content was higher in biopsies classified as Ishak stage 6 than in those classified as Ishak stage 5 (Figure 1C), although the difference in median content (independent samples median test, *P* = 0.27) or distribution (Mann–Whitney *U*‐test, *P* = 0.064) was not significant. There was a wide range of elastin content in biopsies from both Ishak categories: Ishak stage 5, median content of 2.51% (IQR 2.07–4.36; minimum of 1.44, maximum of 8.05); and Ishak stage 6, median content of 3.61% (IQR 2.65–4.52; minimum of 1.69, maximum of 11.19).

**Table 1:** Clinical, biochemical and histological characteristics of patients of the Trent HCV study cohort

Variable	Median	IQR	Number
Age (years)	49	42–54	30
Male gender			23 (77%)
BMI (kg/m^2^)	27	25–30	27
Estimated duration from infection to biopsy (years)	28	20–31	27
Past heavy alcohol use			17 (57%)
Heavy alcohol use (>50 units/week)			9 (30%)
HCV genotype			
1			10 (33%)
2			0
3			17 (57%)
4			1 (3%)
Albumin (g/l)	36	38–40	27
Bilirubin (μmol/ml)	12	9–16	29
ALP (u/l)	113	86–220	29
GGT (u/l)	166	82–339	29
ALT (u/l)	127	85–201	29
Ishak stage 5			16 (53%)
Ishak grade			
0–6			13 (43%)
7–12			16 (53%)
13–18			1 (3%)
Laennec stage			
3			3 (10%)
4A			3 (10%)
4B			10 (33%)
4C			14 (47%)
PSR (collagen) (%)	22.8	15.6–29.9	30
Elastin (%)	3.4	2.2–4.7	30
Biopsy length (mm)	14.5	11.8–18.0	
Number of portal tracts	15	13–20	
ALBI score	−2.6	−2.77 to −2.39	27

ALBI, albumin–bilirubin; ALP, alkaline phosphatase; ALT, alanine transaminase; BMI, body mass index; GGT, γ‐glutamyl transferase; HCV, hepatitis C virus; IQR, interquartile range; PSR, picrosirius red.

The difference in PSR content between Ishak stages 5 and 6 was significant (Figure 1D; 18.80% ± 11.52% versus 30.57% ± 11.24%; Welch two‐sample *t*‐test, *t* = –3.46, degrees of freedom = 42.46, *P* = 0.0012). There was a statistically significant, moderate positive correlation between elastin content and collagen content (Figure 1E; *r*_s_ = 0.58, *P* = 0.000047).

**Figure 2:**
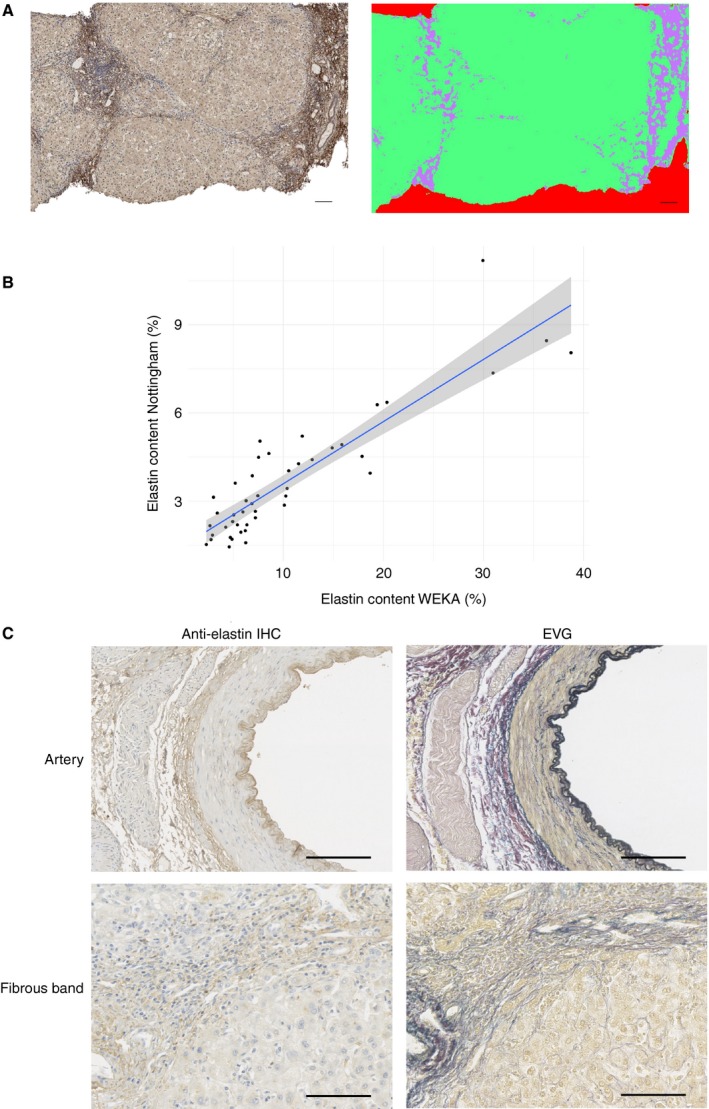
Independent validation of immunohistochemical elastin quantification and technical validation. Elastin contents in original whole‐slide images were independently quantified with an alternative machine learning‐based approach (**A**) to classify images into elastin‐immunopositive (lilac) and elastin‐immunonegative (green) tissue or blank (red) pixels. There was very strong rank correlation of elastin quantified with both methods (**B**,* r*_s_ = 0.87, *P* < 0.00001). Explant liver from a second liver centre from cases with a spectrum of primary aetiologies was independently stained with a different batch of the same primary anti‐elastin antibody by use of a non‐automated protocol to demonstrate centre‐agnostic and disease‐agnostic applicability (**C**, representative images); sections from the same blocks were also tinctorially stained to identify elastin (Elastic van Gieson) and demonstrate spatial equivalence in areas of fibrosis and internal elastic lamina of arteries as an internal positive control. Scale bars: 100 μm.

For technical validation of elastin staining and quantification, elastin was quantified from the same whole‐slide images by an independent observer blind to all data using an alternative method of image classification (weka). There was very strong rank correlation of elastin quantified with both methods (Figure 2A,B; *r*_s_ = 0.87, *P* < 0.00001). However, as expected from the raw values, absolute agreement between methods showed systematic proportional differences on the BA plot (Figure S1), and poor agreement by ICC [one‐way; ICC(1) = 0.098, *P* = 0.258].

To confirm the reproducibility of immunohistochemical detection of elastin in fibrotic liver and supportive tinctorial staining, sections from native explant hepatectomies, from a spectrum of primary aetiologies, and from a single non‐fibrotic partial hepatectomy were obtained at a second UK liver centre and stained in a different laboratory with a separate batch of the same primary antibody. A manual protocol that did not require an automated stainer, with alternative signal amplification, was used. In parallel, tinctorial identification of elastin by EVG staining was undertaken on sections from the same block (Figure 2C) to confirm elastin identification.

Elastin was evident and quantified in fibrous scars of cirrhotic explants (median cirrhotic elastin content of 4.7%, IQR 2.2–9.3; non‐fibrotic elastin content of 0.2%; Figure S2), with clear histological spatial equivalence between elastin immunopositivity and dark blue/black elastin tinctorial staining by EVG. Staining intensity was reduced as compared with automated staining. This demonstrated broad aetiology‐agnostic relevance and the interlaboratory applicability of antibody‐based image analysis to quantify elastin.

### Elastin content can predict adverse clinical outcome in patients with advanced fibrosis

Seventeen patients (57%) progressed to a liver‐related clinical outcome in the follow‐up period after liver biopsy (median follow‐up of 5.8 years; IQR 3.0–8.3). Recorded outcomes were: ascites (*n* = 8), variceal bleeding (*n* = 1), liver‐related death (*n* = 2), and HCC (*n* = 5); one patient presented with ascites and encephalopathy simultaneously. None of the patients underwent transplantation.

Factors associated with time to subsequent clinical outcomes were determined (Table 2). On Cox regression analysis, only elastin content and alkaline phosphatase (ALP) were significant in univariate analysis; Ishak stage and PSR (collagen) content were not predictors of adverse outcome within this cohort. ALP remained significant in multivariate analysis [ALP hazard ratio 1.009, 95% confidence interval (CI) 1.0033–1.015, *P* = 0.0021; elastin hazard ratio 1.123, 95% CI 0.891–1.402, *P* = 0.306].

**Table 2:** Predictors of liver‐related clinical outcomes; elastin content and serum alkaline phosphatase (ALP) are the only significant predictors of a liver‐related clinical event, as determined by univariate Cox regression analysis

Variable	Exp(*B*)	95% CI	*P*‐value
Age (years)	1.059	0.989–1.134	0.100
Male gender	0.405	0.137–1.198	0.103
BMI (kg/m^2^)	1.093	0.961–1.245	0.177
Estimated duration from infection to biopsy (years)	1.011	0.955–1.071	0.704
Past heavy alcohol use	1.371	0.464–4.049	0.568
Heavy alcohol use (>50 units/week)	1.980	0.707–5.544	0.194
Genotype^a^	1.889	0.588–6.070	0.286
Albumin (g/l)	0.947	0.868–1.034	0.947
Bilirubin (μmol/ml)	1.031	0.960–1.107	0.404
ALP (u/l)	1.010	1.004–1.015	0.001
GGT (u/l)	1.001	0.999–1.003	0.279
ALT (u/l)	0.994	0.987–1.001	0.088
Ishak stage^b^	1.307	0.500–3.415	0.584
Laennec stage 3 (reference)			
4A	0.490	0.030–7.961	0.616
4B	0.661	0.068–6.399	0.721
4C	2.058	0.265–16.005	0.490
Ishak grade, 0–6 versus 7–18	0.890	0.334–2.372	0.816
PSR (collagen) (%)	1.010	0.947–1.078	0.758
Elastin (%)	1.226	1.007–1.493	0.042
ALBI score	1.836	0.759–4.439	0.178

ALBI, albumin–bilirubin; ALT, alanine transaminase; BMI, body mass index; CI, confidence interval; GGT, γ‐glutamyl transferase; PSR, picrosirius red.

a Genotype 1 used as reference; analysis performed with comparison of only genotypes 1 and 3.

b Ishak stage 5 used as reference standard.

**Figure 3:**
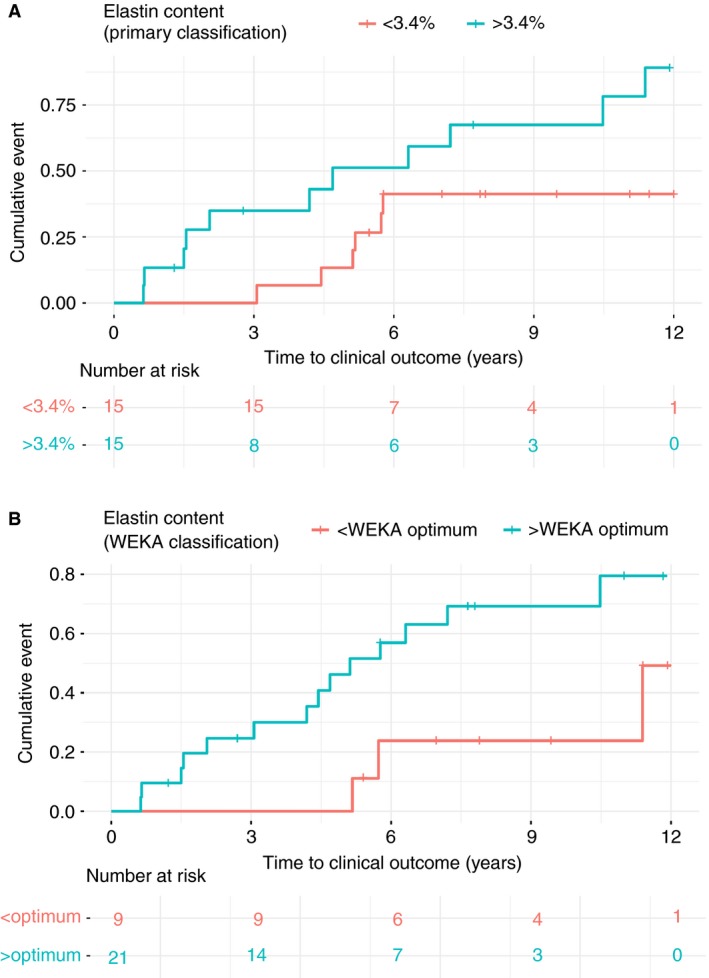
Elastin content functions as a tissue biomarker predictive of adverse liver‐related events. **A**, Patients whose liver biopsies had elastin content greater than the median (3.4%) according to primary classification more rapidly developed an adverse liver‐related event than those with biopsies whose elastin content was below this value; the difference in time to outcomes was significant (log rank: chi‐square 3.98; *P* = 0.046). **B**, weka quantification indicated that biopsies with elastin content greater than the optimum calculated cut‐off (6.2%) more rapidly developed an adverse liver‐related event than those with elastin content below this value; the difference in time to outcomes was significant (log rank: chi‐square 4.60; *P* = 0.0312).

The median elastin content determined with the primary quantification method for the cohort was 3.4% (IQR 2.2–4.7). Eleven of 15 patients (73%) with greater than median elastin content progressed to a clinical outcome, as compared with only six of 15 (40%) of those with elastin content below the group median. The difference in time to outcomes was significant (log rank: chi‐square test 3.98; *P* = 0.046; Figure 3A). Elastin content quantified by weka classification (median 7.5, IQR 5.9–14.4) was also predictive of clinical outcome; a calculated optimum cut‐off value of 6.2% also gave a significant difference in time to outcome (log rank: chi‐square test 4.6; *P* = 0.0312; Figure 3B).

We performed a subgroup analysis to ascertain whether elastin was a predictor of HCC as a single outcome in our cohort. Only five patients (17%) developed HCC during the follow‐up period. Elastin content was not statistically associated with the development of HCC [Cox regression: exp(*B*) 1.00 (0.61–1.62); *P* = 0.99].

## Discussion

In this study, we have developed a method to obtain clinically useful prognostic information from existing liver biopsy material, using an anti‐elastin primary antibody and digital image analysis to detect and quantify elastin content in advanced CLD, and confirmed with independent classification methods. Histological equivalence in identification of elastin by both immunohistochemistry and a well‐characterized tinctorial stain (EVG) was demonstrated. The use of an antibody to identify elastin, based on initial animal studies by the Edinburgh group,^18^, ^32^ has the advantage of conferring the specificity that may be needed in antifibrotic studies, but the disadvantage of adding additional complexity and cost as compared with tinctorial stains.

As a proof‐of‐concept, we chose a group of patients with HCV infection and advanced CLD lacking a sustained virological response to standard contempory treatment (i.e. pegylated interferon and ribavirin). This restricted cohort was chosen to allow the study of patients with rapid fibrosis progression and the highest rate of adverse clinical outcomes. A recent study demonstrated that elastin content is associated with HCC development^21^ in patients with advanced fibrosis. However, this was over a limited follow‐up period, and elastin as a predictor of decompensated disease was not evaluated. We demonstrated significant variation in elastin content in patients with advanced CLD, and showed that elastin content can be used as a tissue biomarker of adverse liver‐related outcomes. In contrast, neither Ishak stage nor PSR (collagen) content predicted outcome in this cohort.

Liver biopsy remains an important clinical diagnostic tool,^3^ but there is a clear obligation to extract as much information from biopsy material as possible, given the associated risks.^1^ The data provide encouragement to examine elastin in large, prospective cohorts of advanced liver fibrosis patients with continuing liver injury. The demonstration of elastin in different aetiologies of human disease suggests its potential utility across all CLDs, but this requires evaluation in disease‐specific cohorts.

Staging of fibrosis with available ordinal scoring systems is subject to considerable observer variability and generates crude ordinal data, indicating a need for alternative methods of evaluation. Quantification of fibrosis from biopsy material by the use of PSR‐stained sections addresses some of these problems, but is not routinely undertaken in clinical practice. In our study, establishment of a laboratory‐specific standard allowed elastin content to be used as a tissue biomarker predicting individual outcome from the clinically indicated biopsy with minimal operator input once standards had been established. With minimal cost and from a standard, clinically indicated liver biopsy, quantification of elastin could be incorporated as part of more nuanced histological assessment, e.g. stratifying patients who are unlikely to benefit from putative antifibrotic therapy, or identifying those requiring earlier referral for assessment for liver transplantation. The ability to obtain this information from whole‐slide images, which can be centrally verified and easily accessed, is of additional value to clinical trials wishing to ensure that appropriate patients are included for investigation of novel therapies.

The resistance of hepatic elastin to degradation suggests that elastin content may influence the balance of fibrogenesis and reversibility in favour of scar accumulation. Progressive scar formation with consequent bridging of vascular structures leads to portal hypertension. The non‐HCC adverse liver‐related outcomes in this proof‐of‐concept study are related to portal hypertension. Increased hepatic elastin content may be a consequence of aberrant hepatic blood flow and sinusoidal pressure; in the context of liver injury, it has been suggested that elastin is deposited by portal fibroblasts to limit damage caused by increased biliary ductal pressure^33^ and that elastin deposition is a result of tensile and shearing effects.^34^ It is also recognised that a strained ECM leads to greater production of fibrotic glycoproteins than a relaxed ECM.^35^, ^36^, ^37^ Given the focus on predicting clinical outcomes in advanced CLD, the role of elastin in homeostasis or during earlier stages of fibrogenesis has not been examined.

This was an initial proof‐of‐concept study, and therefore has inherent limitations. A single‐aetiology cohort has been studied; there is evidence that matrix composition or amount may vary between diseases,^38^ so further work with additional cohorts of alternative aetiologies and similarly prolonged follow‐up is required. The Cox regression analysis should be interpreted cautiously, given that the size of the pilot cohort studied meant that recognised prognostic factors were not identified as significant by univariate analysis. Additionally, it is clear that interlaboratory differences in staining protocols and performance, as demonstrated by the differences between manual and automated staining in our study, mean that prescriptive application of the cut‐off value derived from this study is inappropriate, and further work to allow laboratory protocol harmonisation would be required before routine application in practice. Quantifying elastin from EVG‐stained sections that are more readily and reproducibly available is an obvious means of minimising interlaboratory staining variation in order to develop a more broadly applicable tool.

Elastin accumulates in fibrotic livers regardless of the underlying aetiology, and is a key determinant of irreversibility; its presence may predict the development of clinical outcomes independently of collagen fibres. Hepatic elastin quantification should be evaluated further in larger studies to establish its potential role in clinical decision‐making and the selection of patients for therapeutic trials.

## Author contributions

T. J. Kendall: acquisition of data; analysis and interpretation of data; drafting of the manuscript; critical revision of the manuscript for important intellectual content; and statistical analysis. G. E. Dolman: study concept and design; acquisition of data; analysis and interpretation of data; drafting of the manuscript; critical revision of the manuscript for important intellectual content; and statistical analysis. C. M. Duff: technical support; and critical revision of the manuscript for important intellectual content. E. C. Paish: acquisition of data; technical support; and critical revision of the manuscript for important intellectual content. A. Zaitoun: acquisition of data; and critical revision of the manuscript for important intellectual content. W. Irving: study concept and design; and critical revision of the manuscript for important intellectual content. J. A. Fallowfield: analysis and interpretation of data; drafting of the manuscript; and critical revision of the manuscript for important intellectual content. I. N. Guha: study concept and design; analysis and interpretation of data; drafting of the manuscript; critical revision of the manuscript for important intellectual content; and study supervision.

## Conflicts of interest

J. A. Fallowfield has received consultancy fees from Novartis and Merck, and research grant funding from GlaxoSmithKline and Intercept Pharmaceuticals for work unconnected with that reported in this article. The other authors state that they have no conflicts of interest.

## Supporting information


**Figure S1.** Bland–Altman plot comparing elastin quantification by primary and weka methods.

**Figure S2.** Elastin contents of cirrhotic explant and non‐fibrotic resection cases, determined by weka quantification after laboratory‐specific classifier training.
