# Supplementary figures and images for: Hepatic elastin content is predictive of adverse outcome in advanced fibrotic liver disease
Source: Histopathology. 2018 Apr 16;73(1):90–100. doi: 10.1111/his.13499 (PMC6033111; doi:10.1111/his.13499)

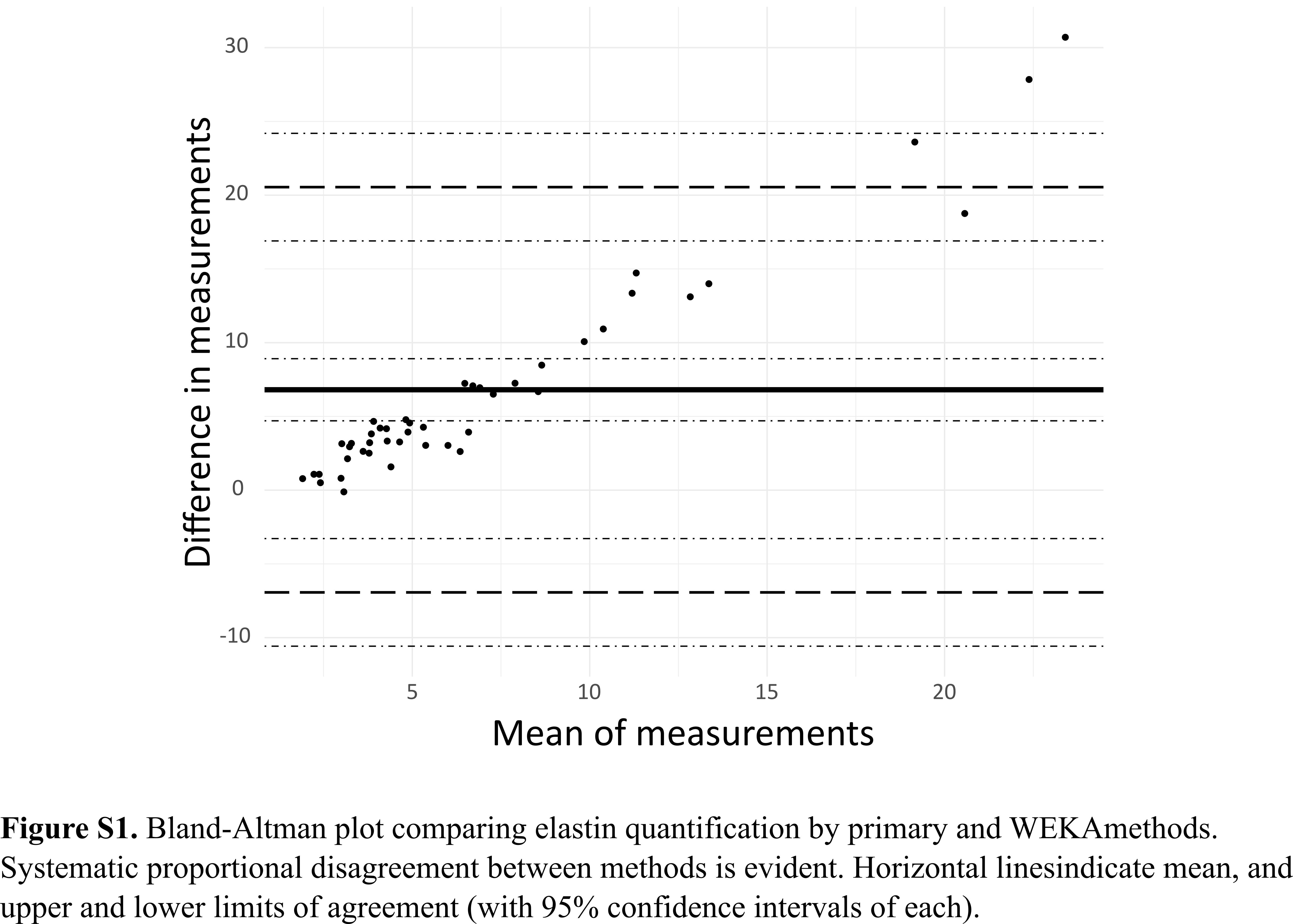

Supplement: Supplementary file 1 — Figure S1. Bland–Altman plot comparing elastin quantification by primary and weka methods. [file HIS-73-90-s001.png]

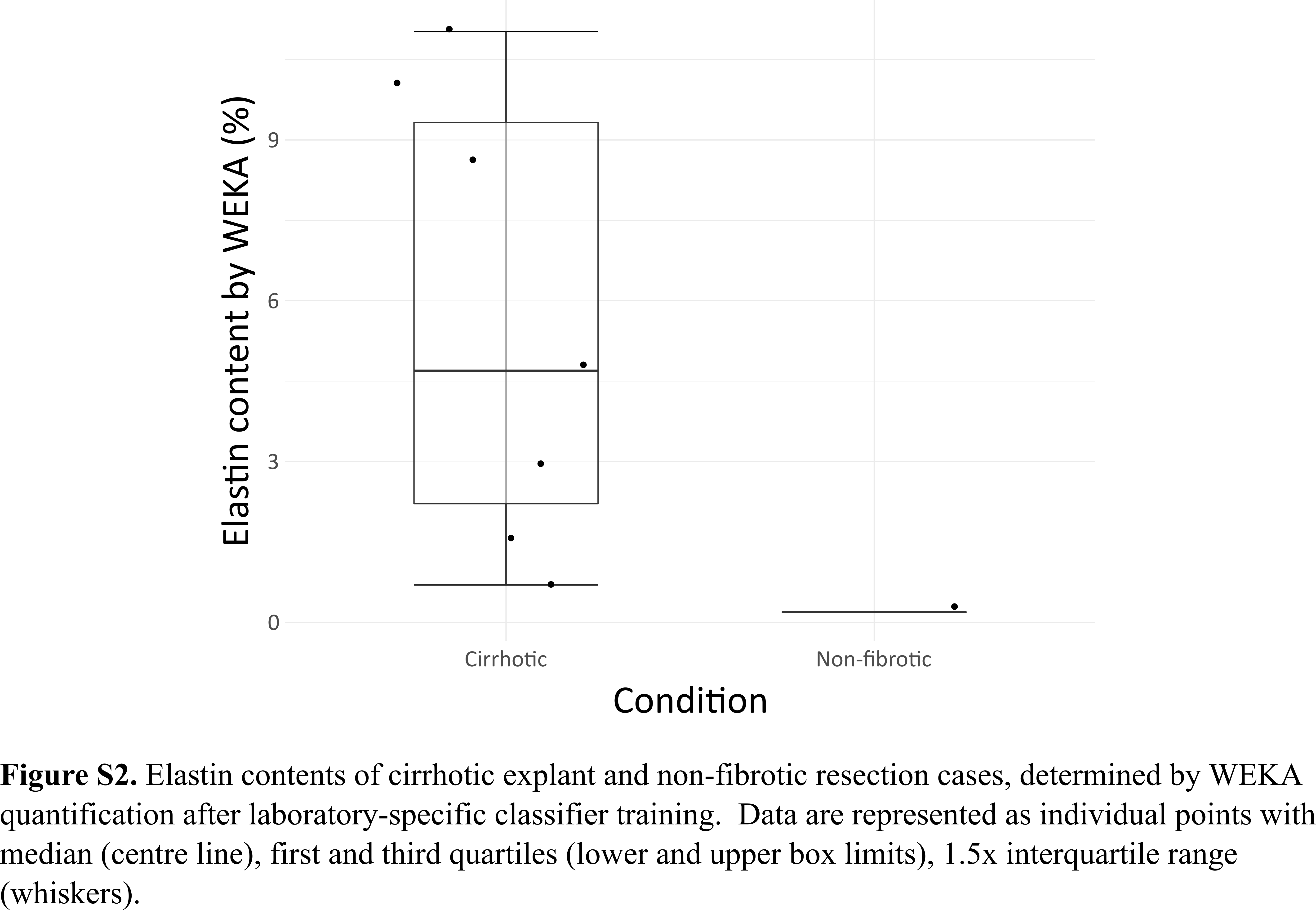

Supplement: Supplementary file 2 — Figure S2. Elastin contents of cirrhotic explant and non‐fibrotic resection cases, determined by weka quantification after laboratory‐specific classifier training. [file HIS-73-90-s002.png]
